# Ohio Veterinary College

**Published:** 1894-03

**Authors:** W. F. Fox

**Affiliations:** Notary Public in and for Hamilton County, Ohio; Cincinnati


### COMMUNICATION.

Ohio Veterinary College.

Cincinnati, Feb. 22d, 1894.

Ed. Archives:—It is with no small degree of regret that the writer offers this manuscript for publication; especially regretting that the cause or causes leading to it are of a more or less personal nature, and necessitating in rebuttal that which may *seem* (but is not) laudation of the subject in question, viz.: the Ohio Veterinary College.

This institution was established some three years or more ago by capitalists of this city, who stand among the highest as citizens of honorable repute; they are men who would blush at the thought of having lent support to an unworthy object. Like all similar institutions it had to cope with difficulties, and probably made some mistakes; the profession at large thought the Deanship was in bad hands, and after two sessions the aforesaid principal was discharged, the school reorganized and more thoroughly equipped, *at the request of the Directors*, who have not, and do not now desire to make it an institution for profit; they *are not* begging students, nor do they want those who do not feel that they will personally profit by their attendance.

Now, the institution has been grossly censured without cause, statements having been made and heralded in print that have no foundation whatever.

The writer read the statements of a member at the 1893 meeting of N. Y. State Society, some of which were true, but in the main were unjust and unworthy the voice of a professional gentleman. Yet, thinking these statements were made unthoughtedly, and would probably be retracted upon investigation, we patiently awaited the call meeting of same Society for January, 1894. But lo! according to the report in Archives, no effort had been made to learn the facts concerning this college, and the rash statements made by certain members were more astoundingly unfounded and malicious than at the preceding meeting.

The reflection cast upon the Faculty by one gentleman we would deem intolerable in the presence of a body of professional men such as compose the N. Y. Association. This person, Dr. Wende, is connected with an institution for which we have the greatest respect, and for this reason could not refer to him as he has unjustly referred to us; we can say this, that the principal of the college for which he so zealously—but maliciously—applies himself is too honorable and just to even sanction or approve of the unwarranted references and assertions made by Dr. Wende.

The statements are too plainly but the overflow of personal malice to be of any damage to our school.

We court investigation, and will not be found wanting. A few plain facts to which affidavit has been duly made, will not be out of place :

1—The institution is not for profit, and its highest aims are for the elevation of the profession.

2—No student has been accepted or allowed to enter the senior course who has not attended one full course here or elsewhere, and no student has ever graduated here who had attended less than the prescribed course, viz.: *two sessions, six months each.*

3—Its Directors are men of wealth and social standing in the community.

4—The Faculty is composed of twelve instructors or professors, five of whom are graduate veterinary surgeons; their professional standing needs no comment, and any fair-minded person can look them up and be convinced that as teachers and lecturers—*as a Faculty*—they are on an equality with that of any similar school.

5—Students must have attained the age of twenty-one years prior to receiving the degree of Doctor of Veterinary Science.

6—That although the assertions of opposers are too absurd to prove damaging to the institution, we yet feel, because of our confidence in the leaders of the N. Y. Association, that those statements not only should be but will be retracted; for, although we have no desire to monopolize or "wipe from the face of the earth" all similar schools, we do propose to forever continue and to grow —not in point of attendance, but in the power of imparting knowledge and broadening the capabilities of those who favor us with their attendance.

7—The students of this college positively assert themselves as thoroughly satisfied, and further proclaim that they have had exceptional advantages while here for acquiring a proper knowledge of their chosen profession. All we desire is justice, and our opposers should remember *abussus non tollit usum.*

STATE OF OHIO,<br>HAMILTON CO. } *ss.*

Neil B. Jones, author of the foregoing letter, on oath, says that the facts and things set forth in the above letter are true, as he verily believes.

NEIL B. JONES, Principal.

Sworn to and subscribed before me this 23d day of February, 1894.

W. F. FOX,
Notary Public in and for Hamilton County, Ohio.

---

### SIXTH INTERNATIONAL CONGRESS OF VETERINARY MEDICINE.

The announcement has just been received fixing the meeting of the Sixth International Congress of Veterinary Medicine in Switzerland, 1895. The Veterinary Societies throughout the United States should take immediate action and prepare to have this country properly represented. The following is the letter of information :

"*To the Veterinary Colleges, Professional Societies, and to all Veterinarians of Every Country.*

"GENTLEMEN AND HONORED COLLEAGUES :—At the close of the Fifth International Congress of Veterinary Medicine in 1889, it was decided that the next Congress should be held in 1894, in Switzerland, and that its organization was to be left in the hands of the Swiss members of the Fifth Congress.

"Following a report made by one of the members to the Swiss government, it has been decided that the Sixth International Veterinary Congress shall be held at Berne (Switzerland) in 1895, and the following committee was appointed: President, Colonel Potterat, chief army veterinarian ; First Vice-President, Prof. H. Berdez, Director of the Veterinary School of Berne ; Second Vice-President, Prof. Hirzel, President of the Swiss Veterinary Society at Zurich ; Secretary, Professor Noyer, of the School of Berne ; Members, Messrs. Schindler of Glaris ; Suter, of Diertal ; Gillurd, of Locle ; Knusel, of Lucorne, and Beretta, of Lugano."

